# Annexin A1 and Autoimmunity: From Basic Science to Clinical Applications

**DOI:** 10.3390/ijms19051348

**Published:** 2018-05-03

**Authors:** Maurizio Bruschi, Andrea Petretto, Augusto Vaglio, Laura Santucci, Giovanni Candiano, Gian Marco Ghiggeri

**Affiliations:** 1 Laboratory of Molecular Nephrology, Istituto Giannina Gaslini, Largo Gaslini n 5, 16147 Genoa, Italy; mauriziobruschi@gaslini.org (M.B.); laurasantucci@gaslini.org (L.S.); giovannicandiano@gaslini.org (G.C.); 2 Core Facilities-Proteomics Laboratory, Istituto Giannina Gaslini, Largo Gaslini n 5, 16147 Genoa, Italy; andreapetretto@gaslini.org; 3 Nephrology Unit, University Hospital, University of Parma, Viale Gramsci n 14, 43100 Parma, Italy; augusto.vaglio@virgilio.it; 4 Division of Nephrology, Dialysis, and Transplantation, Scientific Institute for Research and Health Care (IRCCS), Istituto Giannina Gaslini, Largo Gaslini n 5, 16148 Genoa, Italy

**Keywords:** systemic lupus erythematosus, autoimmunity, lupus nephritis, Annexin A1, Neutrophil Extracellular Traps

## Abstract

Annexin A1 is a protein with multifunctional roles in innate and adaptive immunity mainly devoted to the regulation of inflammatory cells and the resolution of inflammation. Most of the data regarding Annexin A1 roles in immunity derive from cell studies and from mice models lacking Annexin A1 for genetic manipulation (Annexin A1^−/−^); only a few studies sought to define how Annexin A1 is involved in human diseases. High levels of anti-Annexin A1 autoantibodies have been reported in systemic lupus erythematosus (SLE), suggesting this protein is implicated in auto-immunity. Here, we reviewed the evidence available for an association of anti-Annexin A1 autoantibodies and SLE manifestations, in particular in those cases complicated by lupus nephritis. New studies show that serum levels of Annexin A1 are increased in patients presenting renal complications of SLE, but this increment does not correlate with circulating anti-Annexin A1 autoantibodies. On the other hand, high circulating Annexin A1 levels cannot explain per se the development of autoantibodies since post-translational modifications are necessary to make a protein immunogenic. A hypothesis is presented here and discussed regarding the possibility that Annexin A1 undergoes post-translational modifications as a part of neutrophil extracellular traps (NETs) that are produced in response to viral, bacterial, and/or inflammatory triggers. In particular, focus is on the process of citrullination of Annexin A1, which takes place within NETs and that mimics, to some extent, other autoimmune conditions, such as rheumatoid arthritis, that are characterized by the presence of anti-citrullinated peptides in circulation. The description of pathologic pathways leading to modification of Annexin A1 as a trigger of autoimmunity is a cognitive evolution, but requires more experimental data before becoming a solid concept for explaining autoimmunity in human beings.

## 1. Annexin A1: Structure, Cell Expression, and Functions

Annexin A1 is a 37 KDa protein belonging to the annexins superfamily that includes 13 molecules with structural similarities and Ca^2+^-dependent phospholipid–binding properties [1] (mainly related to Phospholipase A2); annexins regulate, in this way, eicosanoid generation. Annexins have a core of four similar repeats in common while the site of specificity is the N-terminus [1]. Annexin A1 is expressed in the cytoplasm of several peripheral blood cells, mainly in neutrophils, monocytes, macrophages, eosinophils, mast cells, and in minimal amounts in T-cells [2,3]. Annexin A1 is detectable in several tissues (the highest levels in seminal fluid) and also in serum. It has multifunctional roles in the frame of control and resolution of inflammation [4,5]. Upon cell activation the protein moves from the storage sites and translocates to the membrane and it is then secreted following different, and cell-specific, pathways [6,7]. It is important, in this context, that Annexin A1 levels incirculating neutrophils are under the control of glucocorticoids (endogenous and exogenous) that involves the Annexin A1 Lipoxin A receptor (ALXR), the glucocorticod-induced leucine zipper gene (*GILZ*) [8], and probably other cytokines, such as Interleukin 6 (IL6) [9]. Regulation of innate and adaptive immunity, and more in general of inflammation, is not the topic of this review, but a summary may help the comprehension of Annexin A1 implication in autoimmunity.

### 1.1. Regulation of Inflammatory Cells

Annexin A1 is a key negative regulator of innate immunity; neutrophils are a major target of this activity [9]. Inhibition by Annexin A1 of transmigration and recruitment of these cells at the inflammatory site has been shown by in vivo experiments in mice models [10]. By analogy, in the presence of Annexin A1, neutrophils have decreased adhesion to endothelial cells in vitro [11]. The opposite occurs in the absence of Annexin A1, such as in the case of *Annexin A1*^−/−^ cells which show increased transmigration across the same cell monolayers and present more chemotaxis in response to inflammatory stimuli [12]. The development of *Annexin A1*^−/−^ mice [13] has contributed to extend the knowledge on Annexin A1 biology leading to the conclusion that, in the absence of Annexin A1, inflammation is prolonged and its negative consequences are exacerbated. *Annexin A1*^−/−^ mice, for example, develop more severe inflammatory lesions of cartilages in experimental models of arthritis [14,15]. By analogy, in experimental models of stroke and bowel diseases, *Annexin A1*^−/−^ mice develop, respectively, more severe neurological problems and have a difficult repair of colitic lesions [16]. Overall, the findings above strengthen the concept that Annexin A1 contributes to restricting the process of recruitment of neutrophils during the development of the inflammatory response and counteracts, in this way, their pro-inflammatory activity as an early event of innate immunity. Lack of inhibition at this step produces accelerated and severe organ lesions.

### 1.2. Resolution of Inflammation

Neutrophil apoptosis and removal by macrophages is functional to reduce thetime of antigen presentation and limits, in this way, the activation of the adaptive system. Annexin A1 modulates all the cell events above [17,18]. First, it induces neutrophil apoptosis [19] by promoting calcium flux and by inhibiting antagonists of the pro-apoptotic factor B-cell lymphoma 2 (BCL-2) [17]. A second main function is to act as a chemoactant for monocytes that remove necrotic debris [20]; this reduces the time of exposure of molecules that potentially stimulate the autoimmune system. A third effect of Annexin A1 is to reduce the activation of pro-inflammatory Toll-like receptors in dendritic cells. Inthe presence of Annexin A1, dendritic cells display a tolerogenic habitus characterized by reduced Cluster of differentiation (CD80), CD86, and Cluster of differentiation (MHC) class II expression and low secretion of Tumor necrosis factor (TNF) and IL12. It is of interest that in such a condition the expression of the inhibitory Programmed death-ligand (PDL1) (which is involved in self-tolerance) is maintained; this means that dendritic cells are tolerogenic within a non-danger context [21].

### 1.3.Regulation of T Cells

Annexin A1 effects on T cells are still debated and many conclusions on the interconnection between Annexin A1 and adaptive immunity are still under way. One reason for the lack of agreement on this point is that T cells express low levels of Annexin A1 [22], albeit this expression is up-regulated during inflammation [23]. Moreover, in standing conditions, the expression of the Annexin A1 receptor ALXR by T cells is also negligible [9] and increases after stimulation. In vitro studies demonstrated that exogenous Annexin A1 enhances T cell receptor signaling and transcription after T cell stimulation [24]; Akt and Extracellular-regulated kinase (ERK) phosphorylation are activated in this context. It seems that Annexin A1 promotes Th1-cells (via IL2 and interferon γ) and suppresses a Th2-cell profile mediated by several cytokines [25]. This effect has important rebounds in animal models of arthritis and, in particular, in the collagen-induced model, in which case, administration of Annexin A1 exacerbates joint inflammation linked with the increased release of the Th1 cytokine, Interferon gamma (IFN-γ).

### 1.4. The Net Balance of Annexin A1 Effects

From what was described above it appears that Annexin A1 may play an anti-inflammatory effect in the context of innate immunity by inhibiting transmigration of neutrophils towards an inflammatory site. Glucocorticoids play their anti-inflammatory effects prevalently at this step by regulating transmigration of neutrophils mediated by Annexin A1. In conditions in which up-regulation by corticosteroids of Annexin A1 is missed, such as in mice knocked-out for the glucocoticoid-induced leucine zipper gene (*GILZ*^−/−^) [8], neutrophil migration is not inhibited. On the other hand, Annexin A1 stimulates Tcell differentiation in Th1 and increases IFN-γ synthesis playing, in this context, a pro-inflammatory effect. Modulation of Tcell receptor strength and, more in general, Tcell activation are epiphenomena of the pro-inflammatory effect. Between the two, i.e., anti- and pro-inflammatory roles, Annexin A1 also plays a tolarogenic effect within dendritic cells that seems to be considered protective of any autoimmunity activation. The existence of anti-Annexin A1 antibodies in systemic lupus erhytematosus (see below) represents the final aspect of the different activities of Annexin A1 in human beings. We should start from this finding and attempt an evaluation of any factor that could influence autoimmunity. First of all, we need to know if free Annexin A1 circulating levels vary in concomitance with anti-Annexin A1 antibodies and try to outline any clinical correlations among free Annexin A1, antibodies, and other clinical determinants.

## 2. Annexin A1 in Experimental and Human Rheumatoid Arthritis

Rheumatoid arthritis is one of the earliest inflammatory condition in which the role of Annexin A1 has been investigated. Actually, Rheumatoid arthritis is to be considered a clinical-pathologic entity midway between inflammation and autoimmunity. In fact, besides a clear inflammatory activity involving several joints, rheumatoid arthritis (RA) is characterized by the presence of antibodies versus citrullinated α-enolase, theformation of which is triggered by mimicry with a conserved sequence on citrullinated enolase of P. gengivalis responsible of periodontitis [26,27]. Several models of experimental AR have been utilized to expand the knowledge on functions of Annexin A1 in vivo. Studies in human beings are rare. In animal models, Annexin A1 has been reported to be both protective and pro-active. Several studies support the former possibility (i.e., the anti-inflammatory function) according to which deficiency of Annexin A1 or depletion by specific antibodies, abrogates the inhibitory effects of glucocorticoids on cytokines and chemokines implying that glucocorticoids require Annexin A1 for their anti-inflammatory effects [15,28,29]. On the other hand, *Annexin A1^−/−^* mice present higher susceptibility to collagen-induced arthritis compared to wild-type mice, implying that the direct role of this protein is protective in respect to articular pathology lesions [29]. By contrast, studies in the same model of collagen-induced rheumatoid arthritis demonstrated that exogenous Annexin A1 worsens the severity of articular lesions [24].

Studies in human beings are merely descriptive. Recent results demonstrated increased Annexin A1 in peripheral CD4^+^ T cells in patients with Rheumatoid arthritis and decreased the response to methyl-prednisolone [30]. In synovial fibroblasts, Annexin A1 expression is, instead, increased in association with steroid therapy and follows local cytokines’ over-production [31]. Taken together, the observations above suggest that Annexin A1 is more dynamically modified in response to the inflammatory milieu in tissues than in circulation, but we lack studies that define correlations between local and/or peripheral Annexin A1 and clinical parameters.

## 3. Anti-Annexin A1 Antibodies: What Is Known

### 3.1. General Considerations

Anti-lipocortin 1 *Immunoglobulin* M (IgM) antibodies were first described in the serum of patients with SLE and with rheumatic arthritis by Goulding and colleagues in 1989 [32]. At the time, the homology between Lipocortin-1 and Annexin A1 was unknown. In the original study, only patients with rheumatoid arthritis who had received steroids for a long period had increased levels ofanti-Lipocortin-1 IgM antibodies, whereas this association was not reported for SLE patients. In the same study, anti-Lipocortin-1 *Immunoglobulin G* (IgG) levels in patients with SLE and/or Rheumatic arthritis were reported as normal suggesting that the IgM profile would result from repetitive antigen challenging. After the identification of Lipocortin-1 as Annexin A1 in 1994, a few other studies confirmed the presence of anti-Annexin A1 antibodies in SLE [33], in particular, in patients with skin lesions [34]. Later on, high levels of anti-Annexin A1 antibodies were reported in association with renal complications [35]. The later condition (i.e., SLE with a renal phenotype) is of main clinical importance since lupus nephritis occurs in almost 40% of SLE patients and represents the most serious complications, potentially leading to chronic renal failure and death [36,37].

### 3.2. Discoid Lupus

Kretz et al. [34] first described the association of anti-Annexin A1 antibodies with cutaneous lupus erythematosus in a large population of patients and, in particular, in those with discoid lesions. Serum anti-Annexin A1 IgG and IgM were subsequently studied in two small cohorts of Chinese patients with SLE (with and without skin lesions) by Meng et al. [38] who, however, did not confirm the original finding and reported higher levels of both anti-Annexin A1 IgG and IgM in healthy controls than in SLE. The different ethnicity of the populations studied, and technical problems related to enzyme-linked immunosorbent assay (ELISA), limit the value of the studies above and do not allow a conclusion on any association of anti-Annexin A1 antibodies with cutaneous lupus.

### 3.3. Lupus Nephritis

**Figure 1:**
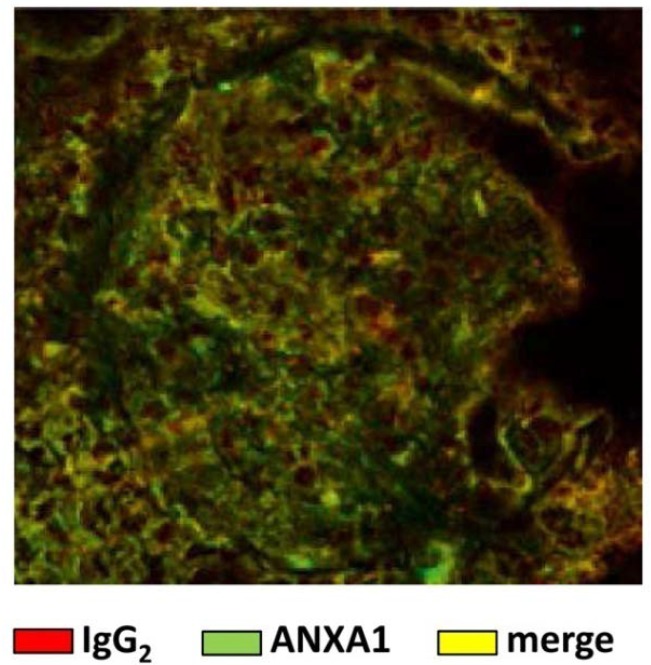
Immunofluorescence of a glomerulus in a renal biopsy of a patient with lupus nephritis. In red are stained IgG2 and in green Annexin A1. A diffuse merge (in orange) indicates the presence of antibodies of IgG2 isotype against Annexin A1.

The association of anti-Annexin A1 and SLE has been recently reported by our group [35,39,40]. We described the presence of circulating auto-antibodies of IgG2 isotype versus Annexin A1 in a high percent of SLE patients and also suggested that these antibodies represent a specific characteristic of lupus nephritis. Renal autoantibody deposition in the glomeruli of the same patients have been shown as well (Figure 1). The novelty of the finding above was that the IgG2 isotype of anti-Annexin A1 auto-antibodies has been proposed as a fingerprint of SLE [40]. The mechanism(s) leading to autoantibody formation in SLE and, in particular, the reason for the IgG2 switching are not clear yet; in the following sections a unifying hypothesis for a common route of generation that includes the formation of anti-DNA IgG2 and anti-αenolase IgG2 will be proposed.

## 4. Anti-Annexin A1 Antibodies: What Is New

**Figure 2:**
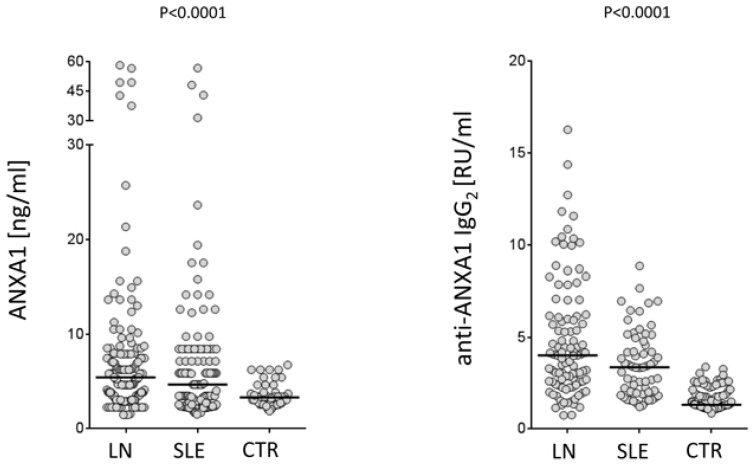
Serum levels of free Annexin A1 and anti-Annexin A1 IgG2 were determined in the two cohorts of patients with SLE, lupus nephritis and controls who were recruited in this study. Levels were determined by specific ELISAs. Overall 216 patients, i.e., 113 with incident SLE and 103 with incident LN, and 50 healthy donors were studied. In both cohorts, females prevail over males (103/13 and 90/13, respectively) and both had a median age of 35 years. Sera were obtained from all patients at the time of diagnosis when steroids were already started in 65% of SLE and 76% of LN patients. Results were given as the median and inter-quartile range.

**Figure 3:**
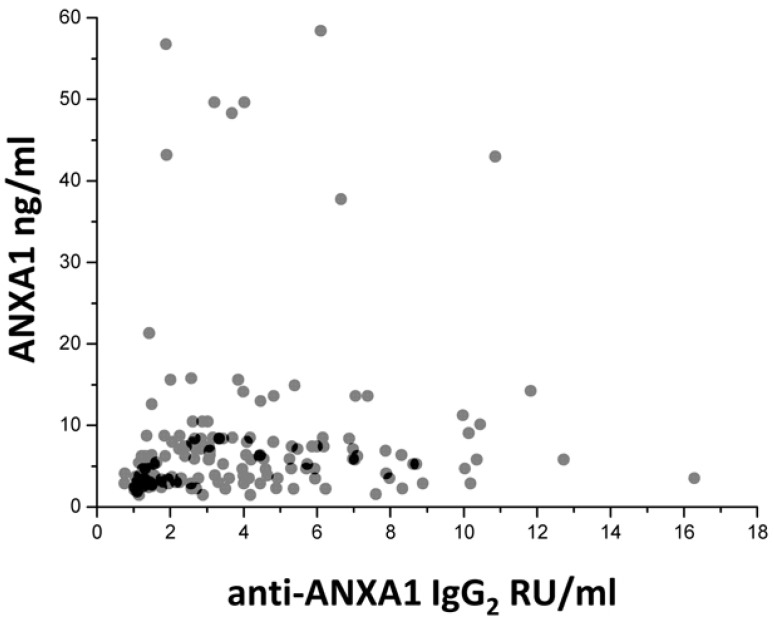
Lack of correlation between serum levels of Annexin A1 and circulating anti-Annexin A1 antibodies in the two cohorts of SLE patients with (gray circles) and without (black circles) lupus nephritis.

Recent studies in our laboratory addressed some of the key points relative of Annexin A1 involvement in autoimmunity and, specifically, in SLE and lupus nephrites. We had the opportunity to study a large cohort of patients with SLE recruited within a nationwide database (the Zeus study, https://clinicaltrials.gov; study number: NCT02403115). Serum levels of both free Annexin A1 and anti-Annexin A1 antibodies were determined in a relevant cohort of 219 patients, 103 of which presented incipient lupus nephritis at their recruitment. Some of the new findings that are presented here represent the basis for evolving in the understanding of mechanisms for developing auto-antibodies in SLE. The pathogenesis of the disease is, in fact, strictly dependent on autoantibodies and, therefore, any effort devoted to clarify their generation would help the comprehension of the disease. A first point is that Annexin A1 serum levels are high in patients with SLE and, in particular, in those with lupus nephritis compared to healthy controls matched for age and sex (Figure 2). This is the first study reporting serum levels of Annexin A1 in SLE and, for this reason, this finding cannot be compared with other databases. Anti-Annexin A1 is, in parallel, higher in patients with lupus nephritis compared to both SLE and controls (Figure 2). It is of some interest that circulating anti-Annexin A1 IgG2 and free Annexin A1 levels are not correlated with each other (Figure 3), nor anti-Annexin A1 IgG2 correlated with serum markers of SLE (i.e., *Complement* component 3 (C3), *Complement* component 4 (C4), pathology class, etc.) and with clinical parameters (including SLE activity, corticosteroid doses, etc.); a unique correlation was found between circulating anti-Annexin A1 IgG2 and anti-ds DNA antibodies that are one of the key markers of SLE activity. Overall, the data on circulating levels of anti-Annexin A1 IgG2 confirm and extend previous reports [39,40]. A lack of correlation between free Annexin A1 and anti-Annexin A1 autoantibodies suggests that the mechanism for autoimmunity does not involve circulating Annexin A1 per se, nor is it correlated with the immune-modulating functions that the protein may play in this context (see above sections on Annexin A1 functions). This key finding on a parallel increment of free Annexin A1 levels and of anti-Annexin A1 antibodies and the lack of correlations among the two, probably closes the debate, lasting for many years, on the possibility that antibodies block Annexin A1 effects and that this block is involved in determining autoimmunity. The conclusion here is that the two phenomena are unrelated.

In general, the formation of auto-antibodies versus a protein should involve post-translational structural changes and, therefore, a lack of correlation of autoantibody with Annexin A1 levels is not surprising. We require studies that focus on structural aspects of Annexin A1 in different settings that involve the possibility that Annexin A1 could be present outside the cells in aggregate with other proteins. This is a topic in rapid evolution, since data from the literature indicate that aggregates of DNA and proteins are extruded from the cells and that this mechanism is involved in autoimmunity. Circulating neutrophils are involved in this context and the formation of neutrophil extracellular traps, or NETs, is the result of neutrophil activation.

## 5. Modifications of Annexin A1 for Autoimmunity

The formation of NETs represents a phase of innate immunity in which neutrophils extrude their own DNA to form a net for entrapping bacteria and viruses [41,42]. It is a sort of premature cell death that starts with de-condensation and release of nuclear chromatin outside the cell and leads to the formation of a physical net where pathogens are entrapped and killed by elastase, defensin, ROS, Myeloperoxidase (MPO), etc. [41,43,44]. The production of superoxide oxygen by Nicotinamide adenine dinucleotide phosphate (NADPH) oxidase constitutes the biochemical culprit of Neutrophil Extracellular Traps (NET) osis [45]; its activation is followed by a cascade involving several kinases downstream of PKC (i.e., c-Raf, MEK, Akt, and ERK) [46,47,48]. Activation of neutrophil-elastase and myeloperoxidase is the second event in NETosis; they dissemble F-actin, de-condense chromatin, and destroy membranes allowing DNA to be released outside the cell [49,50]. The expression of modified DNA and of other post-translational modified components of the nucleosome is a characteristic of NETs [51,52,53,54]. Formation of specific auto-antibodies in other autoimmune conditions, such as in small vessel vasculitis, has been already explained on the basis of the idea that antigens of Cytoplasmic antineutrophil cytoplasmic antibodies (cANCA) (i.e., proteinase 3) and of *Perinuclear Anti-Neutrophil cytoplasmic antibodies* (pANCA) (i.e., MPO) are exposed through NETs to the environment [55,56,57]. More in general, it is now believed that NETs serve as source of modified antigens for ANCA and represent a main driver of auto-immunity in small vessel vasculitis [58,59,60,61]. NETs formation is also increased in SLE [62,63,64,65].

Bruschi and col. (Mn submitted) made a detailed characterization of the protein composition of NETs purified from serum and/or produced ex vivo by neutrophils obtained from patients with SLE, lupus nephritis and controls. Annexin A1 was found to be a main component of NETs, in particular in patients with lupus nephritis who over-expressed this protein. Fine proteomic characterization showed that Annexin A1 had undergone Arginine188 deimination with the formation of citrulline, a process also known as citrullination. This is of interest since citrullination of arginine in other proteins is considered a process leading to autoimmunity (see below). Deimination of arginine is induced by peptidyl arginine deiminase4 (PAD4), a key enzyme in the process of decondensation of nuclear chromatin. Actually, PAD4 deiminates several arginines in histone 3, a stabilizer of the nucleosome backbone, and produces steric modification of the binding with Heterochromatin binding protein 1 (HBPβ1 the final result is de-stabilization of heterochromatin [66,67]. Following deimination of arginines, nucleosome becomes more fluid and DNA and histones are extruded from cells. In this way, DNA, histones, and other proteins are exposed to the environment where, due to post-transductional changes, they are potentially recognized as non-self (see below) and activate the formation of anti-DNA and anti-histone antibodies in circulation.

Several citrullinated autoantigens that are targets of autoantibodies in several autoimmune conditions have been recognized [68] and Annexin A1 is only the last of a list that has been composed over the years. Rheumatoid arthritis is the most studied clinical condition in which citrullinated arginines have been recognized; studies performed in the last few years have shownan association of antibodies versus α-enolase citrullinated at the peptide 5-21 (CEI-1) with periodontitis [26,27] and the joint pathology typical of this disease. Citrullination of α-enolase preferentially occurs in smokers who have an Human leukocyte antigen beta subunit (HLA-DRB1) [69,70].

Therefore, the finding of citrullination of Arginine 188 in Annexin A1 in NETs is consistent with what is believed to be pertinent to brake tolerance and induce auto-immunity in rheumatoid arthritis. At this stage, this is only a working hypothesis that requires experimental evidence to be sustained.

## 6. Anti-Annexin A1 IgG2 Specific Isotype

**Figure 4:**
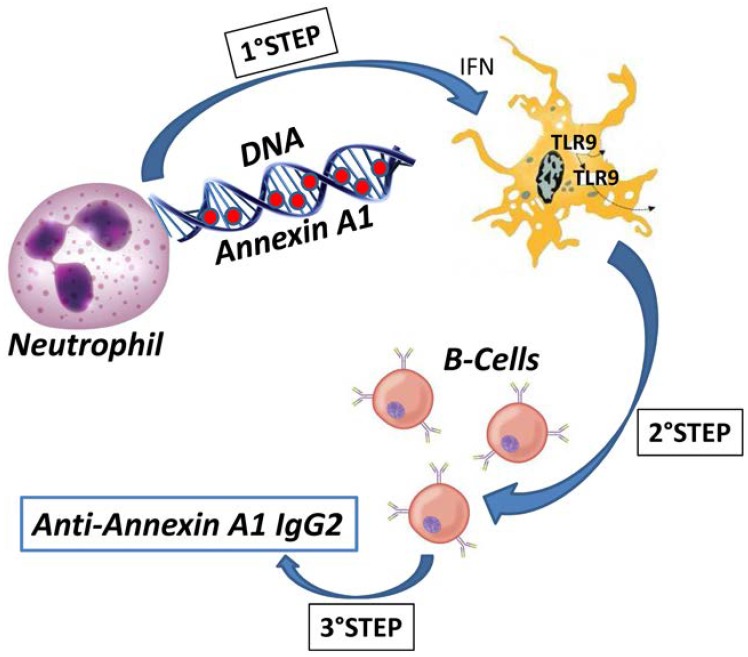
This cartoon shows a potential route for generation of anti-Annexin A1 antibodies of IgG2 isotype in Lupus nephritis. In a first step, deiminated (or citrullinated) Annexin A1 is extruded from neutrophils and it is exposed within neutrophil extracellular traps (NETs). In a second step, modified Annexin A1 is recognized by TLR9 with the intervention of IFN. The final step is the production of anti-Annexin A1 IgG2 by B cells.

The IgG2 isotype of anti-Annexin A1 antibodies in SLE/lupus nephritits is in agreement with the concept that SLE and lupus nephritis are two autoimmune conditions characterized by isotype specificity of auto-antibodies [35,40] and differ from other autoimmune conditions that are instead characterized by IgG4 isotype specificity (such as in the case of membranous nephropathy) [71,72] or by IgG3 specificity (such as in the case of autoimmunity associated with infections and cancer) [73]. In lupus nephritis, autoantibodies detected in serum and in glomeruli, including antibodies versus implanted DNA/histones 2A, 3, and 4, are almost uniquely of the IgG2 isotype, with the exception of a few scattered IgG3 anti-DNA and IgG4 for anti-C1q in sub-epithelia membranous deposits of class V lupus nephritits [40]. This suggests different regulatory mechanisms of antibody switching involved in different conditions. The antibody isotype is determined by the heavy chain constant C_H_ region and derives from the isotype switching occurring in mature B cells. It is known that the isotype switching occurs in response to antigen presentation (mainly driven by Toll-like receptors, TLR) and is regulated by the co-stimulatory repertoire. For IgG2, TLR7, and TLR9 that recognize viral/microbial nucleic acids represent the main drivers of antigen presentationtoB cells under interferon α control [74]. Interestingly, specific DNA-peptide complexes in SLE have been shown to trigger innate plasmacytoid dendritic cell activation via TLR9 [75,76] (Figure 4). Exposure to the environment of modified soluble proteins linked with DNA within the extracellular traps of NETs is an excellent model to explain autoimmunity, provided that post-translational modifications, such as in the case of Annexin A1, have occurred. Overall, it seems possible to conclude that the finding of the IgG2 isotype of anti-Annexin A1 antibodies in SLE and lupus nephritis strengthens the general opinion that these two conditions are characterized by antibody specificity. As for the mechanisms, the predominant idea is that TLR7 and TLR9 under the stimulus of IFN [77] are implicated in determining the IgG2 specificity. Overall, we propose a-multi-step-mechanism for the formation of auto-antibodies in SLE and in Lupus nephritis in which auto-antigens are post-translationally modified and exposed to the environment in NETs where they are recognized and exposed to B cells by specialized TLRs that drive the isotype switching from the common IgG3 to IgG2 that is typical of SLE.

## 7. Concluding Remarks

Annexin A1 is a molecule of great interest for its strong immunologic implications. In view of the multiple functions, there is, however, still controversy on which is the resultant of multiple and often contradictory effects. Regulation of inflammatory cells, resolution of inflammation, and activation of Tcells are the major effects of Annexin A1 in the immunologic context. Overall, it is believed that Annexin A1 is an anti-inflammatory molecule playing major role as an inhibitor of neutrophil transmigration and recruitment at inflammatory sites. On the pro-inflammatory side, Annexin A1 promotes T_H_1-cells and suppresses the Th2-cell profile; the final result is the exacerbation of joint inflammation linked with the increased release of IFN-γ. Annexin A1 also modulates adaptive immunity via Tcells and plays tolarogenic functions within dendritic cells. An important feature of Annexin A1 is the correlation with steroids.

The existence of anti-Annexin A1 antibodies in SLE seems a paradox of what is considered above. Circulating anti-Annexin A1 antibodies are high, in particular, in SLE complicated by lupus nephritis and/or in cases with cutaneous localization. In the former case, i.e., in lupus nephritis, in addition to high circulating anti-Annexin A1 antibodies, serum levels of free Annexin A1 are also high, but they do not correlate with each other. Whether high serum Annexin A1 represents a compensatory mechanism to overproduction of antibodies is a matter of concern, but there are several other possible options that could explain this basic finding. On the other hand, the level of circulating anti-Annexin A1 is strongly correlated with other, and more specific, antibodies that are biomarkers of SLE activity, such as anti-dsDNA suggesting, overall, that autoimmunity, in this case, is not correlated with circulating Annexin A1.

New studies also indicate that Annexin A1 in lupus nephritis is a component of NETs that seems of interest in view of the analogy with several other auto-immune conditions, such as small vessel vasculitis, in which components of NETs (i.e., MPO or proteinase 3) serve as antigens for antibodies. Annexin A1 in NETs undergoes post-translational modifications, the majority of which is deimination of Arginine 188 with the formation of citrulline. Future studies would confirm and widen the composition of NETs proteins as triggers of autoimmunity and, more in general, the role of NETs as inducers of auto-antibodies. Basic research and developments in proteomics technology would be complementary in clarifying the missing point of Annexin A1 as an antigen for autoimmunity.
